# Strain engineering of *Aspergillus* species for industrial enzymes bioproduction: a comprehensive review

**DOI:** 10.1007/s44307-026-00140-9

**Published:** 2026-10-30

**Authors:** Irfan Ullah, Zhihui Zhang, Dezhi Geng, Qiyang Zhou, Yahong Zou, Bing Xu, Chong Zhang, Xin-Hui Xing

**Affiliations:** 1 Institute of Biopharmaceutical and Health Engineering, Tsinghua Shenzhen International Graduate School, Shenzhen, 518055 China; 2 Key Laboratory of Active Proteins and Peptides Green Biomanufacturing of Guangdong Higher Education Institutes, Tsinghua Shenzhen International Graduate School, Shenzhen, 518055 China; 3 Foshan Haitian Flavoring & Food Co., Ltd, Foshan, 528000 China; 4 Guangdong Provincial Key Laboratory of Advanced Biofermentation Technology Enterprise in Flavoring & Food, Foshan, 528500 China; 5 Institute of Biochemical Engineering, Department of Chemical Engineering, Tsinghua University, Beijing, 100084 China; 6 Key Laboratory of Industrial Biocatalysis, Ministry of Education, Tsinghua University, Beijing, 100084 China; 7 Center for Synthetic and Systems Biology, Tsinghua University, Beijing, 100084 China; 8 State Key Laboratory of Green Biomanufacturing, Beijing, 100084 China; 9 Beijing Key Laboratory of Recombinant Proteins and Biomanufacturing, Beijing, 100084 China

**Keywords:** *Aspergillus*, Strain Engineering, Industrial Enzyme Production, Fungal Cell Factories

## Abstract

Filamentous fungi of the genus *Aspergillus*, because of their exceptional secretory capacity and metabolic versatility have been extensively applied in traditional food fermentation and industrial biotechnology for production of diverse bioproducts. Their history of domestication and industrial application has enabled major progress in enzyme production; however, challenges associated with the filamentous growth and strain engineering for future biomanufacturing development still remain. This review summarizes the advances across the spectrum of strain-improvement methodologies, from classical mutagenesis, protoplast fusion, genome shuffling, and adaptive laboratory evolution to the modern era of CRISPR enabled genome editing, synthetic biology, and systems-level metabolic engineering. Previous reviews have primarily focused on individual *Aspergillus* species, specific industrial products or particular strain improvement approaches. In this review, we provide a comprehensive overview of classical and modern strain engineering technologies and industrial scale up across a wide range of *Aspergillus* species for industrial enzyme production. In particular, emerging non-GMO evolution tools such as atmospheric and room temperature plasma (ARTP) mutagenesis, and modern molecular biotechnology coupled with microdroplet-based ultrahigh-throughput screening, now allow the researchers to generate and screen a large number of *Aspergillus* species mutants with single-cell precision. The discovery of high-performance strains with enhanced secretion, stress tolerance, substrate utilization, and favorable enzymatic profiles for food and bioprocessing applications has been accelerated by the strain innovations. Collectively, these advances establish *A. oryzae* and the related species as increasingly reliable, scalable, and programmable hosts that can meet the biomanufacturing requirements of future circular and sustainable bioeconomies.

## Introduction

**Fig. 1:**
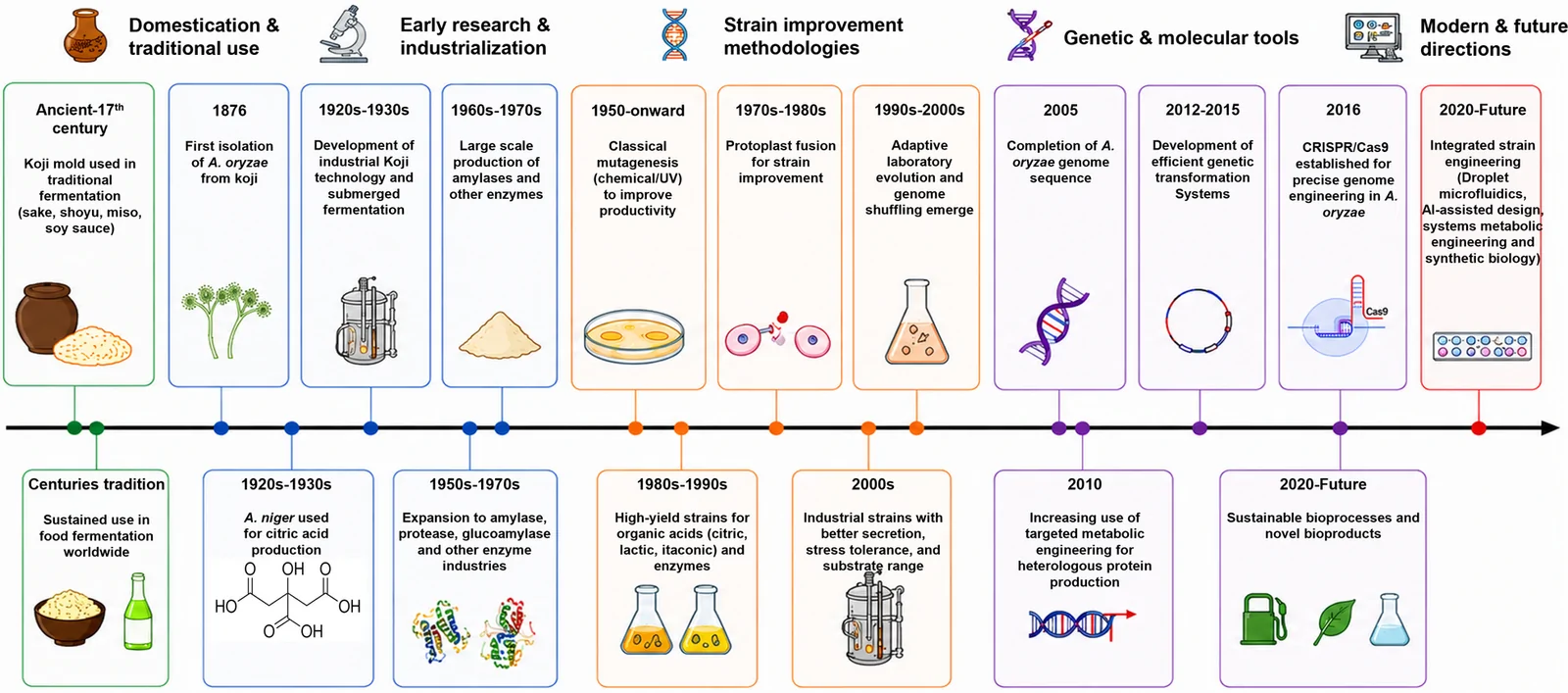
Timeline of key research and application milestones in *Aspergillus* species. The schematic summarizes the developments from traditional food fermentation and early industrialization to classical strain improvement, molecular and genome engineering technologies, and emerging integrated strain engineering approaches. Dates indicate approximate periods associated with the selected milestones and illustrate overall technological progression. Information was compiled from the cited literature (Kitamoto 2015; Li et al. 2023b; Machida et al. 2005, 2008; Nødvig et al. 2015; Papagianni 2007; Parekh et al. 2000; Peberdy 1980; Stephanopoulos 2002). UV: ultraviolet, CRISPR: clustered regularly interspaced short palindromic repeats, AI: artificial intelligence

Filamentous fungi of the genus *Aspergillus* have shaped the foundations of industrial biotechnology for more than a century, serving as one of the earliest and most successful examples of microbial domestication for large-scale bioproduction (Fig. 1). In 1917, James Currie demonstrated *Aspergillus niger* (*A. niger*) as an efficient producer of citric acid, a discovery that laid the foundation of a global fermentation industry (Currie 1917). This innovation had already moved into commercial scale production by 1920, ultimately growing into a multi-billion-dollar sector (Cairns et al. 2018; Latif et al. 2025; Saloheimo 2025; Wollborn 2024). Recent estimates show that enzymes from fungi, especially various *Aspergillus* species such as *Aspergillus oryzae* (*A. oryzae*)*, Aspergillus sojae* (*A. sojae*), *A. niger, Aspergillus flavus* (*A. flavus*)*, Aspergillus fumigatus* (*A. fumigatus*) *and Aspergillus terreus* (*A. terreus*)*,* accounts for a major portion of the industrial enzyme market globally, and continue to grow because of the widespread applications in food, feed, detergent, biofuel and textile industries (Cairns et al. 2018; Meyer et al. 2016). These biotechnological advancements are largely attributed to the exceptional secretory ability of *Aspergillus* species, which naturally produce a diverse range of hydrolytic enzymes essential for biomass biorefinery (Ntana et al. 2020; Rosas-Vega et al. 2025).

The domestication of *Aspergillus* species has occurred in diverse geographical and cultural contexts. In East Asia, specifically in China and Japan, Koji molds like *A. oryzae* and *A. sojae* have been used for hundreds of years for the fermentation of rice, soybeans and wheat into traditional foods such as soy sauce, miso and sake (Gibbons et al. 2012; Liu et al. 2024b). Over successive generations, these strains were selected for improved production of proteases and amylases by solid state fermentation (Watarai et al. 2019). Beyond its traditional applications in sake, miso and soy sauce, *A. oryzae* has undergone significant optimization for industrial food fermentation. Recent developments, specifically those reported by Foshan Haitian Flavoring & Food Co., Ltd., have focused on engineering strains with improved amylase, glutaminase, protease, and aminopeptidase activities for improved fermentation efficiency and flavor quality of soy sauce. Furthermore, mixed strain fermentation enhanced enzyme activity and flavor, demonstrating a shift from traditional strain screening to a function driven breeding of *A. oryzae* for modern food fermentation (Chen et al. 2023; Hong 2024; Huang et al. 2017; Tong et al. 2017a; Wang and Tong 2018; Wu et al. 2024; Zhao et al. 2013b; Zhou and Li 2017). In contrast, Western bio-industrial applications have primarily adopted *A. niger* for biosynthesis of citric acid and enzymes for starch processing such as glucoamylase, which are essential in food fermentation and brewing industries (Cairns et al. 2021). Moreover, other *Aspergillus* species, such as *A. terreus,* are used for the production of lovastatin and itaconic acid, while *A. fumigatus* and *A. nidulans* are used as fungal models (Boruta and Bizukojc 2017). Furthermore, the safety is a critical issue for selection of the fungal species in food industry. *A. oryzae* is classified as Generally Recognized as Safe (GRAS), because it lacks aflatoxin production, while many strains of its close relative *A. flavus* produces cyclopiazonic acid and aflatoxins, making it unsuitable for food applications (Kobayashi et al. 2007). These differences drive modern genome-based efforts to eliminate any residual biosynthetic pathways pertaining to toxin generation from the industrial *Aspergillus* strains (Lehmbeck et al. 2025).

*Aspergillus* species have relatively large genomes (30–37 Mb), enriched with genes for secreted enzymes, carbohydrate active enzymes (CAZymes) and a wide range of biosynthetic gene clusters for secondary metabolites (Kjærbølling et al. 2020; Machida et al. 2005; Theobald et al. 2024). Among them, *A. oryzae* RIB40, a reference strain whose genome was sequenced in 2005 by the Machida group, serves as a model for research (Machida et al. 2005). *A. oryzae* genomic studies showed that it has a wide range of secretory enzymes and genetic clusters for metabolites than its relative filamentous fungi, suggesting its evolutionary adaptations to solid state fermentation (Sato et al. 2011; Watarai et al. 2019). Furthermore, it produces a wide range of extracellular proteins, including several alpha-amylases such as Taka amylase A, proteases, and diverse glycoside hydrolases, which accounts for its strong capacity for the secretion of extracellular enzymes and industrial proteins (Ichishima 2016; Kitamoto 2015). Compared with its closely related species, *A. fumigatus* and *A. nidulans, A. oryzae* encodes significantly different genes for more secreted hydrolases, which further supports its broad biotechnological utility. Therefore, *A. oryzae* and its relative strains have been widely used for the production of glucoamylase, biorefinery enzymes, and heterologous proteins using powerful expression promoters such as *gpdA*, *tef1* and different variants of engineered *AmyB*. Due to its ease of genetic manipulation and GRAS feature, *A. oryzae* has emerged as a promising cell factory for fungal molecular research and industrial biotechnology (Jin et al. 2021; Kitamoto 2015; Sun et al. 2024).

*A. niger* strains likewise show clear lineage specific characteristics shaped by long term domestication and selective breeding for industrial applications. Traditional glucoamylase producing strains have been extensively optimized for efficient enzyme production, acid tolerance and increased productivity, resulting in optimized genetic backgrounds primarily refined through metabolic adjustments and reduction of unwanted byproducts. In contrast, other ‘‘black *aspergilli*’’ such as *Aspergillus luchuensis* (*A. luchuensis*) used in fermentation of *awamori*, have been selected for their ability to produce large amounts of citric acid and adaptation to acidic fermentation environments. Comparative genomic and transcriptomics studies show that the closely related sublineages can differ substantially in their transporters, profiles of secreted enzymes, and regulatory mechanisms. These differences largely depend on their specific domestication history and fermentation conditions (Kobayashi et al. 2007; Sun et al. 2024). Consequently, modern strain engineering strategies increasingly exploit these lineage-specific characteristics through approaches including multicopy *amyB* integration in *A. sojae* and targeted deletion of transcriptional repressors present in wild-type strains (Jin et al. 2021). Overall, comparative strain analyses indicate that the functional differences among closely related *Aspergillus* lineages are driven primarily by variations in gene dosage and regulatory control rather than by the acquisition of entirely novel enzymatic functions (Kjærbølling et al. 2020; Sato et al. 2011).

**Fig. 2:**
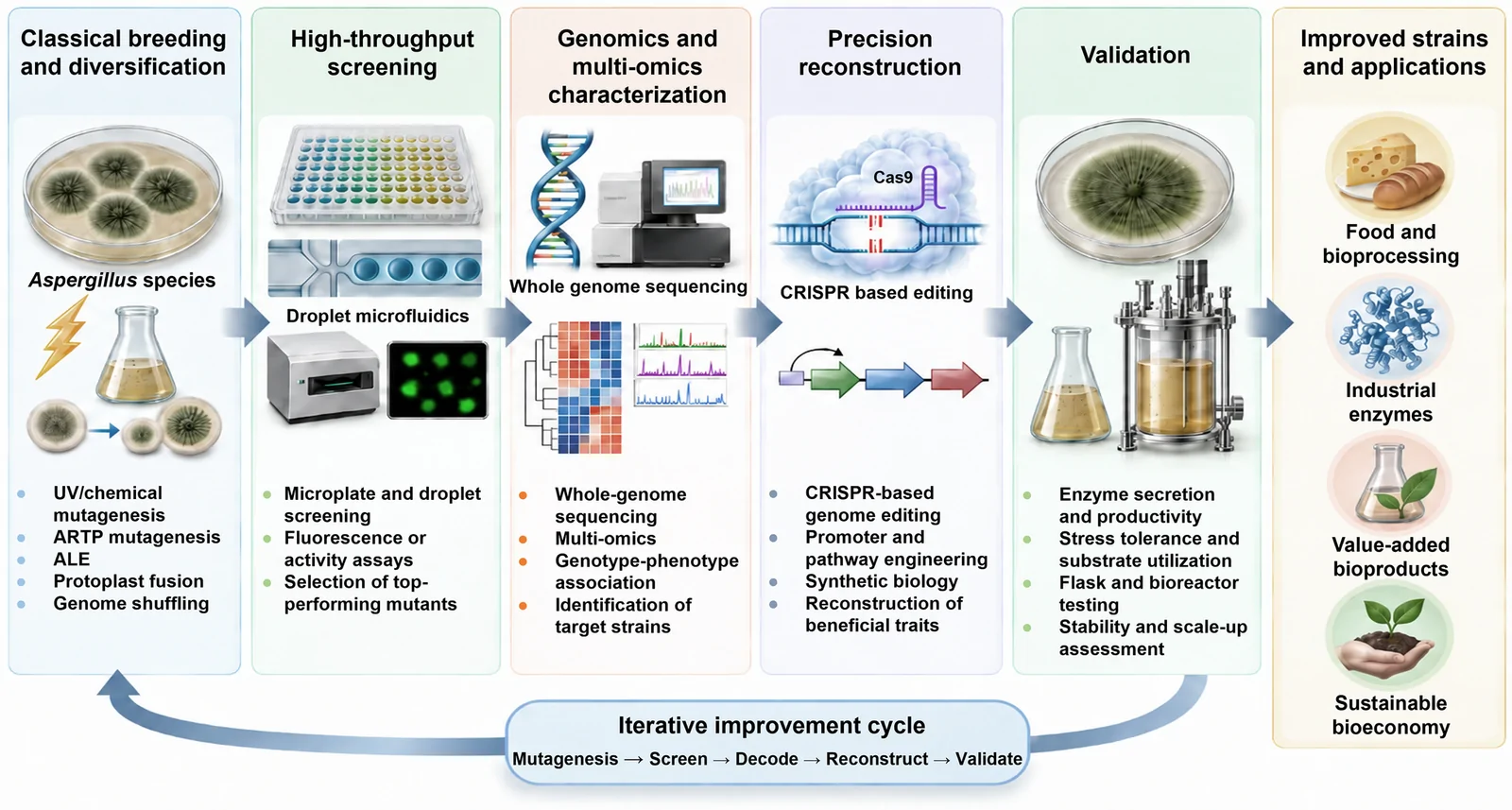
Integrated workflow for *Aspergillus* strain improvement. Classical and evolutionary breeding approaches generate diverse strain populations that are subjected to high throughput screening to identify improved phenotypes. Selected strains are subsequently characterized using genomics and multi-omics approaches, followed by precision reconstruction using genome-editing, pathway engineering, and synthetic biology approaches. Improved strains are then validated for enzyme production, stress tolerance, substrate utilization, genetic stability, and performance under fermentation and scale-up conditions. The superior strains can further be used for iterative strain development cycle towards improved industrial strains and sustainable bioprocessing. UV: ultraviolet, ARTP: atmospheric and room temperature plasma, ALE: adaptive laboratory evolution, CRISPR: clustered regularly interspaced short palindromic repeats

Herein, this review provides an overview of the developments across classical and modern strain improvement strategies, including mutagenesis, protoplast fusion, adaptive laboratory evolution, genome shuffling, CRISPR based editing, synthetic biology frameworks, and emerging high throughput microfluidic screening technologies. Previous reviews have primarily focused on individual *Aspergillus* species, specific industrial products or particular strain improvement approaches. The present review focuses on the comprehensive overview of technological progression and integration of strain improvement strategies across a wide range of industrially relevant *Aspergillus* species with recent advances in high throughput screening for industrial enzyme production. The integrative framework considered in this review links the classical and modern strain improvement with high throughput screening, genomics and multi-omics characterization, and industrial validation and an iterative strain improvement cycle for different enzyme producing *Aspergillus* species (Fig. 2). Through this comprehensive framework, we aim to highlight recent advances, challenges, and future perspectives for chassis development of *Aspergillus* strains for sustainable next-generation green biomanufacturing.

## *Aspergillus* species and their enzymatic repertoires

**Fig. 3:**
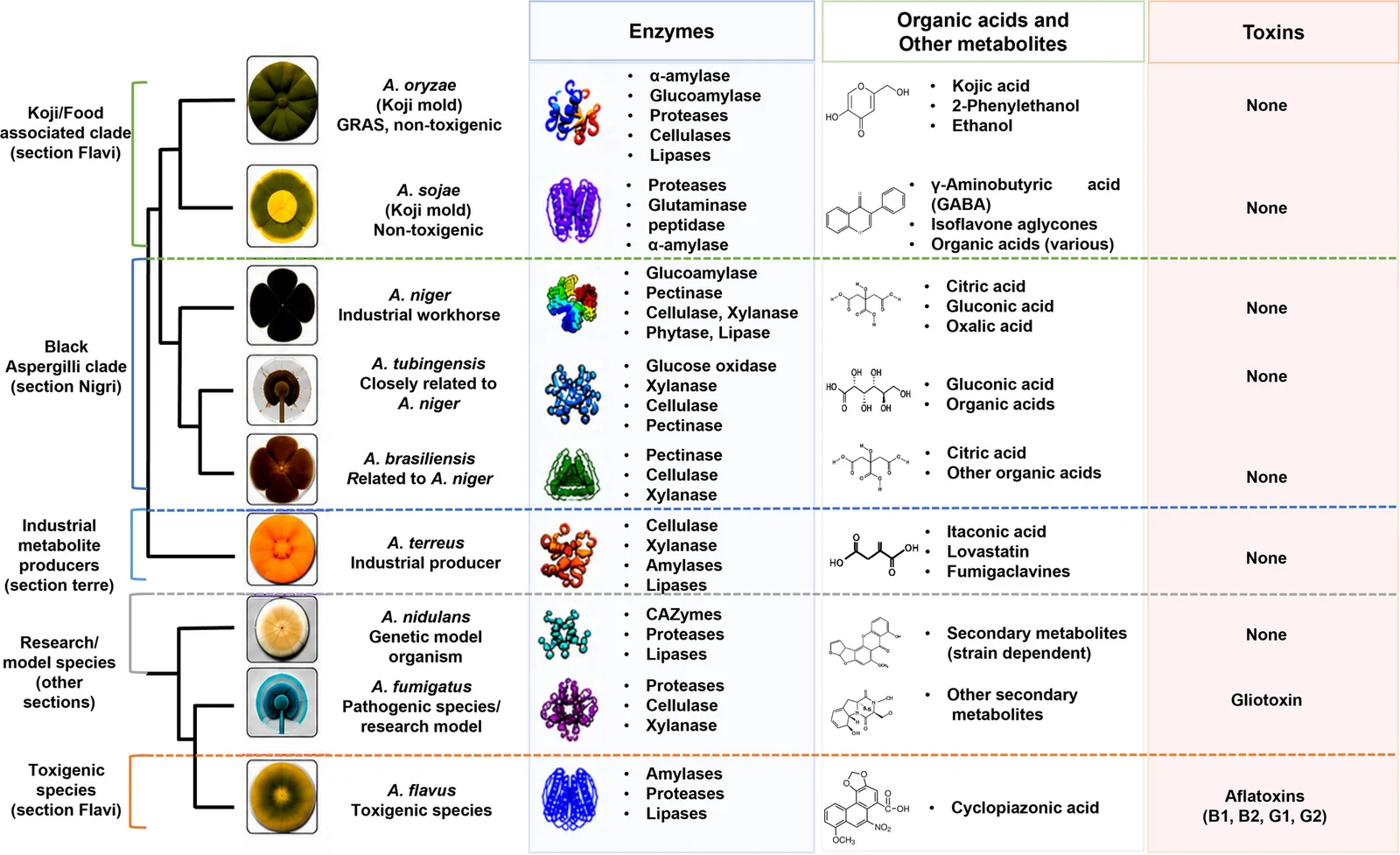
Schematic phylogenetic overview of representative *Aspergillus* species and their major bio-industrial products. The figure summarizes literature-based relationships among selected *Aspergillus* species together with representative enzymes, organic acids and other metabolites, and mycotoxins. Data were compiled from published studies (Cairns et al. 2018; El-Gendi et al. 2021; Kjærbølling et al. 2020; Kobayashi et al. 2007; Lehmbeck et al. 2025; Machida et al. 2005; Sato et al. 2011; Sun et al. 2024; Theobald et al. 2024; Woloshuk et al. 1994). GRAS, generally recognized as safe, CAZymes, carbohydrate active enzymes; GABA, γ-aminobutyric acid

A wide range of *Aspergillus* species function as powerful hosts for industrial enzyme production, each shaped by ecological adaptation, evolutionary divergence, and centuries of human directed domestication (Fig. 3). These fungi possess expansive repertoires of CAZymes, proteases, lipases, and auxiliary metabolic proteins that enable efficient degradation of complex organic substrates (Lehmbeck et al. 2025; Sato et al. 2011). Among these, *A. oryzae*, a domesticated koji mold used in traditional East Asian food fermentations has been extensively selected for strong amylolytic and proteolytic capacities (Acevedo et al. 2025; Ichishima 2016). However, *A. oryzae* retains homologs of the aflatoxin biosynthetic gene cluster which is non-functional in industrial strains due to mutations, deletions and loss of expression of key pathway genes, contributing to its safety status for food applications (Kobayashi et al. 2007; Machida et al. 2005). *A. sojae*, is a domesticated koji mold closely related to members of section Flavi and has been similarly selected for soybean fermentation. Comparative genomic analyses reveal differences in CAZymes between *A. oryzae* and *A. sojae,* which may contribute to their distinct enzyme characteristics (Ito and Matsuyama 2021; Kjærbølling et al. 2020). *A. niger* is one of the most valuable industrial species, producing large amounts of acid stable hydrolases and citric acid, particularly under low pH fermentation environments (Cairns et al. 2018). In addition, it secretes cellulases and hemicellulases naturally, including xylanases and endoglucanases which play an important role in the conversion of biomass, animal feed enhancement and biofuel production (El-Gendi et al. 2021; Rosas-Vega et al. 2025; Wang et al. 2018). These species-specific profiles form the foundation for selecting suitable industrial hosts according to the required biotechnological application.

In *Aspergillus* lineages, domesticated koji molds demonstrate how prolonged selection has influenced the fungal enzyme production and fermentation performance. Although *A. sojae* and *A. oryzae* are closely related in genetics, they have distinct enzyme profiles and appear to have followed different domestication trajectories. Genomic comparisons show that *A. oryzae* is more closely related to *A. aflatoxiformans* or *A. minisclerotigenes* than to *A. flavus*, while the industrial strain *A. sojae* is more closely related to *A. parasiticus* (Kjærbølling et al. 2020). Although both species lack aflatoxin formation, *A. oryzae* retains a wider array of amylolytic genes, which contribute to its superior starch degrading ability. During domestication for enzyme production and food fermentation, *A. oryzae* strains were gradually selected for improved hydrolytic enzyme activity, efficient growth, and improved fermentation performance (Ichishima 2016; Itani et al. 2025). These phenotypes are developed through accumulation of mutations during prolonged strain breeding. While, strains used for miso and soy sauce fermentation have been selected for efficient growth and enzyme production in protein dense environment, and for improved performance under high salt conditions during industrial fermentation, resulting in the improved proteolytic efficiency and tolerance to fermentation stress (Sato et al. 2011; Yao et al. 2023). For example, *A. oryzae* 3.042 (AS3.951) is domesticated in China, and one of the most influential soy sauce strains used in Chinese fermentation industries. This strain was developed through UV mutagenesis during late 1950 s and has been widely adopted nationwide afterwards, which became an industrial standard due to its growth stability, high production of hydrolytic enzyme, and suitable for large scale soy sauce fermentation in China. Unlike other industrial strains limited to individual manufacturers, *A. oryzae* 3.042 achieved nationwide application and played a key role in standardizing Chinese soy sauce production. Furthermore, genome sequencing and comparative genomic analysis revealed different genetic characteristics associated with improved enzyme production, adaptability to environment, and fermentation performance, making this strain a model for understanding the molecular basis of industrial domestication and modern strain development (Shao et al. 2006; Zhao et al. 2012a, 2014a, 2012b; Zhao et al. 2013a). Moreover, recent studies show that differences between domesticated koji molds and their wild type relatives are mainly due to changes in gene copy number, transcriptional regulation, chromosomal rearrangements, and protein secretion pathways that collectively contribute to improved industrial traits (Acevedo et al. 2025; Gibbons et al. 2012). Changes in the copy number, genomic organization and hydrolytic enzyme genes regulation, including glucoamylase and alpha amylase encoding genes, have contributed to improved enzyme production capacity of particular lineages (Kitamoto 2015; Machida et al. 2005). These genomic and physiological adaptations collectively improve protein secretion, substrate utilization efficiency, fermentation robustness, and overall industrial productivity. These findings show how domestication and industrial selection have driven the functional specialization in *Aspergillus* species resulting in distinct lineages which are optimized for specific fermentation and biotechnological applications.

Moreover, other *Aspergillus* species also possess diverse enzymatic profiles with different industrial applications. For example, *A. terreus* is an industrially important species used for the production of lovastatin and has been subjected to strain screening and genetic engineering to improve lovastatin production (Na et al. 2024). Although *A. nidulans* is not commonly used industrially, it remains a key model for fungal genetics, regulatory mechanisms and secondary metabolism (Etxebeste and Espeso 2020; Yaegashi et al. 2014). Comparative genomic analysis across *A. terreus* and related species have revealed significant differences in CAZymes profiles and biomass degradation potential (Theobald et al. 2024; Wu et al. 2023). The diversity of CAZymes profiles among *Aspergillus* species reflects their adaptation to different ecological niches and determines their substrate utilization capabilities for industrial biomass conversion. In addition, genetically engineered strains of *A. nidulans* and *A. fumigatus* have shown strong capabilities for heterologous enzyme production. These species offer effective expression platforms for synthesis of recombinant enzymes, pharmaceuticals and other high value proteins due to their strong secretion systems and well-established genetic techniques (Sun et al. 2024). Collectively, the metabolic diversity of *Aspergillus* species provides a wide range of genetic and biochemical resource for development of specialized microbial cell factories tailored to diverse industrial applications. Table 1 summarizes the key enzymes produced by major *Aspergillus* species, together with genome sequence information that were generated using different strain improvement methods.

**Table 1:** Major industrial enzymes and strain engineering strategies in *Aspergillus* species

Species	Representative genome sequenced strain	Natural enzyme production	Strain improvement techniques applied
*A. oryzae* (Koji mold)	RIB40 (37.9 Mb, NCBI: GCF_000184455.2) (Machida et al. 2005)	α-Amylase, glucoamylase, proteases (acid, neutral, alkaline), lipases, phytases, β-galactosidase, β-glycosidases (Aleem et al. 2018; Ao et al. 2018; Bailey and Linko 1990; Fujita et al. 2003; Gao et al. 2019; Ohnishi et al. 1994; Riou et al. 1998; Salihi et al. 2017)	Classical mutagenesis (UV, N-Methyl-N'-Nitro-N-Nitrosoguanidine (NTG)) (Aleem et al. 2018; Saif-Ur-Rehman et al. 2024), promoter engineering (*amyB*, *glaA*) (Jin et al. 2021), CRISPR-Cas9 genome editing (Chang 2023), protein engineering for enhanced secretion (Sakekar et al. 2021)
*A. niger*	CBS 513.88 (34 Mb, NCBI: GCF_000002855.4) (Pel et al. 2007)	Glucoamylase, pectinases, xylanases, cellulases, phytases, lipases, proteases (Contesini et al. 2017; de Vries and Visser 2001; Pel et al. 2007; Yadav et al. 2020)	Random mutagenesis (Jafari et al. 2017), adaptive laboratory evolution (ALE) (Lubbers et al. 2019; Wang et al. 2023), targeted gene deletions to reduce protease background (Van Den Hombergh et al. 2004), CRISPR-Cas9 genome editing (Jin et al. 2022), synthetic promoter libraries (Zheng et al. 2025), secretory pathway engineering (Gou et al. 2025)
*A. terreus*	NIH2624 (29.3 Mb, NCBI: GCF_000149615.1) (Broad Institute 2006)	Cellulases (Sohail et al. 2016; Theobald et al. 2024), hemicellulases (Ismail et al. 1986), pectinases (Sethi et al. 2016)	Classical mutagenesis (Upendra and Khandelwal 2016), metabolic pathway engineering (Hasan et al. 2021), CRISPR-based modification of central metabolism (Shih et al. 2022)
*A. sojae*	NBRC 4239 (39.7 Mb, NCBI: GCA_000226655.2) (Sato et al. 2011)	Acid proteases (Ito and Matsuyama 2021; Li et al. 2023a), glutaminase (Ito et al. 2013), peptidases (Chien et al. 2002), pectinases (Ustok et al. 2007)	Chemical mutagenesis and irradiation for higher protease titers (Chi and Cho 2014; Lim et al. 2019), response surface optimization (Tari et al. 2007), CRISPR-Cas9 applications (Igarashi et al. 2023)
*A. flavus*	NRRL3357 (37.8 Mb, NCBI: GCA_024678945.1) (Skerker et al. 2021)	Proteases (Wajeeha et al. 2021), lipases (Contesini et al. 2017), amylases (Sundarram and Murthy 2014)	Genetic modification to remove aflatoxin pathway (Yu et al. 2004), strain selection for safe enzyme production (Jun et al. 2022)
*A. nidulans*	FGSC A4 (30.2 Mb, NCBI: GCA_000149205.2) (Galagan et al. 2005)	Wide CAZymes repertoire (de Vries et al. 2017; Rubio et al. 2016)	Genetic model system: homologous recombination (Nayak et al. 2006), CRISPR-Cas9 (Modaffari et al. 2024), synthetic biology toolkits and modular genetic engineering (Nødvig et al. 2018)
*A. fumigatus*	Af293 (29.4 Mb, NCBI: GCF_000002655.1) (Nierman et al. 2005)	Cellulases, xylanases, and chitinases (Nierman et al. 2005)	Gene knockouts to attenuate pathogenicity, CRISPR genome editing (Abdallah et al. 2017; Sugui et al. 2007)
*A. brasiliensis*	CBS 101740 (35.8 Mb, NCBI: GCA_001889945.1) (de Vries et al. 2017)	Xylanases, pectinases, cellulases (Laswai et al. 2024)	UV and chemical mutagenesis (Hooi and Kuan 2015)
*A. tubingensis*, *A. aculeatus*	CBS 134.48 (35.1 Mb, NCBI: GCA_001890745.1) (DOE Joint Genome Institute 2016a) ATCC 16872 (35.4 Mb, NCBI: GCA_001890905.1) (DOE Joint Genome Institute 2016b) (de Vries et al. 2017)	Pectinases, phytases, xylanases, cellulases (Baba et al. 2015; Pandya and Gupte 2012)	Random mutagenesis and targeted deletion of extracellular protease genes (Huang et al. 2019; Xi et al. 2020; Yoshimura et al. 2022)

## Industrial applications of enzymes produced by *Aspergillus* species

**Fig. 4:**
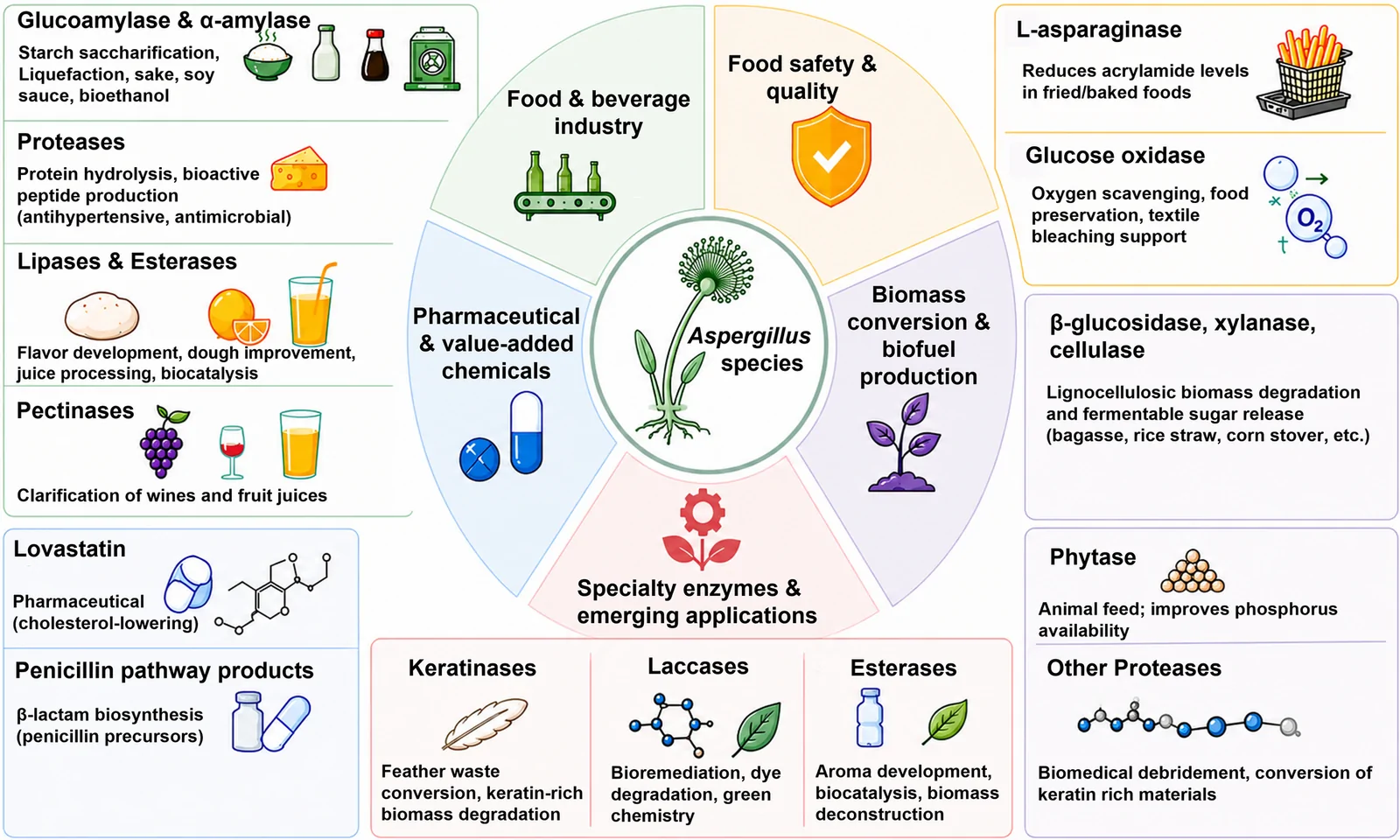
Industrial applications of *Aspergillus* derived enzymes. The figure summarizes representative applications across five major sectors: Food and beverage industry, including glucoamylase, α-amylase, proteases, lipases, esterases, and pectinases; Food safety and quality, including L-asparaginase for acrylamide reduction and glucose oxidase for oxygen scavenging and preservation; Biomass conversion and biofuel production, including β-glucosidase, xylanase, cellulase, phytase, and proteases; Specialty enzymes and emerging applications, including keratinases, laccases, and esterases for waste valorization, bioremediation, green chemistry, and biomass deconstruction; and Pharmaceutical and value added chemicals, including lovastatin and penicillin pathway products. Representative examples were compiled from published studies (Bankar et al. 2009; Jia et al. 2021; Jin et al. 2021; Lei et al. 2013; Marui et al. 2010; Preczeski et al. 2020; Raveendran et al. 2018; Sandri and da Silveira 2018; Temporiti et al. 2022; Thathola et al. 2024)

Enzymes produced by *Aspergillus* species are widely used in food processing, pharmaceuticals, biofuel production and environmental biotechnology because these fungi have high production capacity and produce enzymes with diverse catalytic capabilities (Table 2 and Fig. 4) (Raveendran et al. 2018). In the beverage and food industry, glucoamylases and amylases from *A. oryzae* are widely used in saccharification and liquefaction during production of bioethanol, sake and soy sauce (Kobayashi et al. 2007; Rosas-Vega et al. 2025). Proteases produced by *A. oryzae* and *Aspergillus flavipes* (*A. flavipes*) breakdown milk proteins releasing bioactive peptides with antihypertensive and antimicrobial properties (Zanutto-Elgui et al. 2019). L-asparaginase from *A. oryzae* helps reduce acrylamide formation in fried foods by converting L-asparagine, a key acrylamide precursor, into L-aspartic acid (Dias et al. 2017). In addition, esterases and lipases from *A. niger* contribute to flavor improvement, enhancing texture of dough, and processing of fruit juices, while glucose oxidase is utilized for removing oxygen in textile bleaching and juice preservation (El-Gendi et al. 2021). In conversion of biomass and biofuel production, β-glucosidases, xylanases and cellulases play an important role in lignocellulosic biomass degradation. Different agricultural residues such as bagasse, rice straw and corn stover are treated with fungal enzyme cocktails to break down into fermentable sugars (Rosas-Vega et al. 2025). Among these enzymes, xylanases from *A. niger* have been shown to increase the efficiency of hemicellulose saccharification (Pal and Khanum 2011; Salim et al. 2018). Moreover, phytases produced by *Aspergillus* species improve phosphate bioavailability in animal feed, while pectinases are extensively used in the food industry for clarification of wines and fruit juices using degradation of plant pectins (Lei et al. 2013; Sandri and da Silveira 2018). Representative levels of major industrial enzyme from different *Aspergillus* species together with their production strategies and cultivation conditions, are summarized in Table 3.

**Table 2:** *Aspergillus* species derived enzymes and its industrial applications

Species	Enzymes	Industrial applications
*A. oryzae, A. niger*	Glucoamylase and α-amylase	Starch saccharification, liquefaction, sake, soy sauce, bioethanol (Kobayashi et al. 2007)
*A. oryzae, A. flavipes*	Proteases	Protein hydrolysis, food processing, bioactive peptide production (Zanutto-Elgui et al. 2019)
*A. oryzae, A. terreus*	L-asparaginase	Acrylamide reduction in fried/baked foods (Jia et al. 2021)
*A. niger*	Lipases and esterases	Flavor development, dough improvement, biocatalysis (Contesini et al. 2017; Raveendran et al. 2018)
*A. niger*	Glucose oxidase	Oxygen scavenging, food preservation, textile bleaching support (Bankar et al. 2009)
*A. niger*, *A. oryzae*	β-glucosidase, xylanase, cellulase	Lignocellulosic biomass degradation and fermentable sugar release (de Vries and Visser 2001; Rosas-Vega et al. 2025)
*A. niger*	Phytase	Animal feed; improves phosphorus availability (Lei et al. 2013)
*A. niger*	Pectinase	Clarification of fruit juices and wines (Sandri and da Silveira 2018)
*A. nidulans*	Keratinases	Feather waste conversion, keratin-rich biomass degradation (Preczeski et al. 2020; Saber et al. 2010)
*A. nidulans*	Laccases	Bioremediation, dye degradation, green chemistry (Thathola et al. 2024)
*A. oryzae, A. flavus, A. fumigatus*	Lipases, laccases, esterases	Plastic degradation, sustainable biocatalysis (Temporiti et al. 2022)

**Table 3:** Representative major industrial enzymes and their production levels in *Aspergillus* species

Industrial enzyme	Species / strain	Production strategy / cultivation mode	Reported production level
α-Amylase	*A. oryzae* S2	SmF; Box-Behnken optimization followed by 7L fed-batch cultivation with intermittent starch feeding	1,220 U/mL (Naili et al. 2016)
α-Amylase	*A. niger*	AI-guided fermentation with Raman-based glucose monitoring and closed-loop feeding	15,729.47 U/mL (Wang et al. 2026)
Glucoamylase	*A. niger* BO-1	Industrial strain; fed-batch cultivation	up to 6.5 g/L (Pedersen et al. 2000)
Glucoamylase	*A. niger* AG	*amyA*/*glaA* overexpression combined with 1% casein phosphopeptide supplementation	40,710 U/mL (An et al. 2019)
Acid protease	*A. oryzae* MTCC 5341	SSF on wheat bran; RSM optimization	8.47 × 10^5^ U/g bran (Vishwanatha et al. 2010)
Alkaline protease	*A. oryzae*	SSF using peanut shell; RSM optimization	1,101.778 U/mL (Farooq et al. 2024)
Pectinase	*A. niger*	16L pH-controlled fed-batch with constant sucrose feeding	450 U/mL (El Enshasy et al. 2018)
Phytase	*A. niger* NCIM 563	Statistical medium optimization and scale-up to 2-10L fermenters	164 IU/mL (Shah et al. 2017)
Phytase	*A. oryzae* SBS50	SSF; medium, moisture and nutrient optimization	506.12 U/g DMR (Pragya et al. 2023)
Lipase	*A. niger* G783	SmF; Box–Behnken medium optimization	2,171 ± 41 U/mL (Jia et al. 2015)
β-Glucosidase	*A. niger* CDBB-H-175	SmF; sequential optimization of cultivation conditions and medium composition	1,207.9 U/mL (Alarid-García and Escamilla-Silva 2017)
Cellulase	*A. fumigatus* ABK9	SmF; 2-deoxy-D-glucose mediated catabolite derepression	132.2 U/mL CMCase; 21.3 U/mL FPase (Das et al. 2013)
Xylanase	*A. tubingensis* FAT35	SSF using agro-industrial residues	550 U/mL (Ristović et al. 2026)
Xylanase	*A. fumigatus* NITDGPKA3	SmF using rice straw; Tween-80 supplementation	193.58 IU/mL (Sarkar and Aikat 2014)
Glucose oxidase	*A. niger*	SmF with corn steep liquor; aeration and agitation optimization	850 ± 45 U/mL (Kona et al. 2001)
Feruloyl esterase A	*A. niger*	Homologous overexpression under *gpd* promoter; carbon-source optimization	1.0 g/L (Record et al. 2003)
Feruloyl esterase B	*A. niger* protease deficient strain	Homologous overexpression under *gpd* promoter	~ 100 mg/L (Levasseur et al. 2004)
L-Asparaginase	*A. oryzae* IOC 3999	SmF; statistical medium and cultivation optimization	1,443.57 U/mL (Cunha et al. 2022)
Keratinase	*Aspergillus* sp.	SmF using sterilized chicken feathers	113.50 U/mL (Preczeski et al. 2020)

Production levels are reported in the original units provided by the cited studies. *Abbreviations*: *SmF* submerged fermentation, *SSF* solid-state fermentation, *RSM* response surface methodology, *DMR* dry matter residue, *CMCase* carboxymethyl cellulase activity, *FPase* filter paper activity, *U* enzyme activity unit, *IU* international unit

In addition to hydrolytic enzymes, *Aspergillus* species play an important role in producing value added chemicals and pharmaceuticals. Lovastatin and itaconic acid produced by *A. terreus* have broad applications in the polymer industry and pharmaceutical (Boruta and Bizukojc 2017). Similarly, engineered strains of *A. oryzae* have been developed for the production of penicillin precursors (Marui et al. 2010). Furthermore, keratinases and proteases are being investigated for biomedical debridement and conversion of keratin rich materials, like feathers. Specialty enzymes such as laccase and cutinases produced by *A. nidulans* and *A. oryzae* are gaining attention for its potential applications in green synthetic chemistry and bioremediation (Preczeski et al. 2020; Temporiti et al. 2022).

Despite substantial laboratory scale advancement, engineered *A. oryzae* implementation in industrial settings face different challenges including limited oxygen transfer, shear force sensitivity, pellet formation, and mixing constraints caused by viscosity during fermentation (Sun et al. 2024). Therefore, industrial scalability needs to be considered from an early stage of strain engineering. Future strategies should combine rheology optimization, morphology engineering and increased tolerance to stress to create strains that are capable of high production under industrial continuous and fed batch fermentation conditions. Therefore, next generation strain modifications should prioritize not only achieving high enzyme productivities, but also long-term durability and industrial scalability (Wollborn 2024).

## Classical and evolutionary strain improvement methods

Classical strain improvement strategies formed the backbone of *Aspergillus* biotechnology long before molecular genetics matured. For more than five decades, classical strain improvement has served as the primary strategy for industrial strain development and established the foundation for many of the commercial enzyme producing strains used today (Meyer et al. 2020; Salazar-Cerezo et al. 2023). Random mutagenesis using Ultraviolet (UV) irradiation, Ethyl methane sulfonate (EMS), N-methyl-N-nitro-N-nitrosoguanidine (NTG/MNNG), X‑ray exposure and the newly emerging atmospheric and room temperature plasma (ARTP) mutagenesis; has been used to enhance hydrolase secretion, stress tolerance, organic acid production, and growth performance (Lim et al. 2019; Nemoto et al. 2009). Mutagenesis efforts in *A. oryzae*, *A. niger*, and *A. terreus* produced strains with enhanced production of acid protease, kojic acid, cellulase, and amylase titers, some exceeding several fold improvements (Aleem et al. 2018; Saif-Ur-Rehman et al. 2024). Despite the emergence of precise genome editing technologies, classical mutagenesis remains attractive approach because it is non-GMO, simple and capable of improving many complex traits; however, due to its random mutation pattern it requires extensive screening and subsequent strain stabilization. Currently, mutagenesis is integrated with genome engineering to identify and optimize useful mutations rapidly. Overall, the mutation-based strain improvement remains an essential foundation for industrial biotechnology and continues to complement genome editing techniques by offering significant genetic diversity for subsequent targeted strain engineering.

### UV Irradiation

UV irradiation is a classical strain improvement technique widely used to create improved *Aspergillus* mutants. It mainly causes DNA damage such as pyrimidine dimers which lead to random mutations followed by DNA repair. In *A. oryzae*, UV mutagenesis has been extensively employed to enhance enzyme secretion and heterologous protein production. For instance, *A. oryzae* mutants generated using UV mutagenesis showed improved β-galactosidase production, with mutant UV-15–20 reaching 114.08 U/mL, representing a 49.22% increase compared with the parental strain (Zhang et al. 2014a). Moreover, it has also been applied to increase amylase production in *Aspergillus* and to create hyperproducing mutants such as the AUT1-8 lineage, which produced 2.6-fold more recombinant human lysozyme and 3.2-fold more chymosin than the parent strain through enhanced protein production mechanisms (Kostyleva et al. 2017; Nemoto et al. 2009). Due to its low cost, high efficiency and simplicity, it remains one of the commonly employed mutagenesis methods for *Aspergillus* strain improvement. However, due to its random mutation pattern extensive screening is necessary to identify and maintain desirable mutants (Jin et al. 2021; Nemoto et al. 2009).

### Gamma rays and x-rays

Gamma rays (γ-rays) and X-rays irradiation are classical mutagenesis techniques that have been used to improve industrial *A. oryzae* strains by generating a wide range of genetic variations. Unlike UV irradiation, which induces pyrimidine dimers, γ-rays and X-rays possess greater ability to penetrate and generate a wide range of DNA damage through the generation of reactive oxygen species (Toyoshima et al. 2012). This wide range of mutations increase the chances of obtaining mutants with improved phenotypes. In *A. oryzae*, γ-ray irradiation has been extensively applied for mutagenic breeding and comparative analysis demonstrate that it induces genetic mutations suitable for improving industrial traits. γ-ray irradiation has been successfully applied for *Aspergillus* strain improvement to increase the production of amylolytic enzymes. In *A. oryzae*, γ-ray mutagenesis generated hyperproducing mutants that retained about 95% of their enzyme activity after five generations, exhibiting its effectiveness for generating genetically stable mutants (Kostyleva et al. 2017; Toyoshima et al. 2012). Similarly, it generated *A. niger* mutants with higher glucose oxidase activities of 20.55–27.50 U/mL as compare with its parental strain (7.5 U/mL), demonstrates γ-ray irradiation potential (Zia et al. 2012). Although both methods rely on ionizing radiations, γ-rays mutagenesis has been considerably more widely employed in *Aspergillus* breeding than X-ray irradiation. However, synchrotron soft X-ray irradiation was applied successfully to generate *A. oryzae* mutants resulting enhanced kojic acid production and altered metabolic characteristics (Chen et al. 2006). Due to their ability to generate a wide range of genetic diversity, γ-rays and X-ray mutagenesis strategies are used for developing improved strains with increase enzyme production and fermentation performance. Compared with UV irradiation, γ-rays and X-rays have greater penetration depth and induce diverse genomic mutations; however, they required specialized facilities and exhibit lower mutation efficiency than high linear energy transfer (LET) ion-beam irradiation. Although these mutagenic approaches have been superseded by modern and more efficient mutagenic technologies such as ARTP, they continue to provide useful tools for improvement of industrial strains and functional genetic studies.

### Ion beam irradiation

Ion beam irradiation has emerged as an advanced physical mutagenesis approach for *A. oryzae* due to its high mutation efficiency and unique spectrum of mutation (Satoh and Oono 2019). Unlike traditional UV or γ-rays mutagenesis, high LET ion beam provides dense energy along its irradiation tracks, generating extensive damage to DNA, including chromosomal rearrangements, frameshift mutations, base substitutions. Comparative studies have shown that carbon ion beams exhibit significantly higher lethal mutation effects than γ-rays, generating a wide range of genetic diversity which is important for industrial mutation breeding. Its ability to induce large scale genomic mutations while maintaining higher mutant viability makes ion beam irradiation suitable for improving complex industrial strains. In *A. oryzae*, carbon ion beam irradiation effectively induced selenate resistant mutants and generate a wide range of genetic mutations as compared to γ-rays irradiation, demonstrating its efficiency for generating superior mutants (Toyoshima et al. 2012). In addition, heavy ion irradiation has been used to increase the production of amylolytic enzymes in *A. luchuensis* (Kojo et al. 2017). However, due to requirements for specialized facilities and high operational costs limits its application as compared to UV irradiation and ARTP mutagenesis.

### Emerging new mutagenesis method using atmospheric and room temperature plasma (ARTP) mutation technology

ARTP has recently emerged as an effective non-GMO mutagenesis approach for *Aspergillus* strain improvement by generating genome wide mutations that improve enzyme production and other phenotypic characteristics (Wang et al. 2020; Wu et al. 2026; Zhang et al. 2014b). It generates reactive oxygen species that induce oxidative DNA damage, leading to chromosomal rearrangements, deletions, insertions and base substitutions during DNA repair (Li et al. 2008). It has great potential for microbial breeding which enables the creation of mutant strains with specified features and superior performance, leading to increased titer, yield and productivity, to meet the bio-industrial demands. Compared with UV irradiation and chemical mutagenesis, ARTP provides higher mutagenic efficiency, short treatment times, and enhanced mutant survival, thereby increasing the chances of screening superior mutants (Zhang et al. 2015). In *Aspergillus* species, ARTP mutagenesis has been successfully applied to enhance enzyme secretion, organic acid production, stress tolerance, and metabolic performance through rapid generation of diverse mutant libraries. For example, ARTP mutagenesis improved L-asparaginase production in *A. candidus* and *A. sydowii* by approximately 2.3-fold in genetically stable mutants (Asitok et al. 2023). In *A. oryzae*, ARTP combined with adaptive laboratory evolution (ALE) has also been used to enhance acid protease production and fermentation performance, exhibiting its efficiency for industrial strain breeding. In addition, ARTP-derived mutants have been developed for enhanced production of glucoamylase, cellulases, proteases, lipases, and other industrial enzymes, as well as for improved tolerance to oxidative stress, acidic pH, high substrate concentrations, and fermentation-associated environmental stresses (Ottenheim et al. 2018; Shu et al. 2020). ARTP has also been explored for activation of silent secondary-metabolite pathways and enhancement of bioactive compound production in filamentous fungi (Zhang et al. 2023b). In industrial *Aspergillus* breeding, ARTP is particularly attractive because it is compatible with spores and protoplast-based workflows and can be efficiently integrated with high-throughput screening platforms, including droplet microfluidics and fluorescence-based secretion assays. These combinations of the screening platforms can significantly accelerate the identification of hyperproducing strains for industrial enzyme and metabolite production. Consequently, ARTP has become one of the most important next generation non-GMO mutagenesis techniques for the strain improvement and complements both classical and modern genome engineering strategies (Wu et al. 2017).

### Chemical mutagenesis

Chemical mutagenesis is another widely used classical strain improvement approach for improving strains. Unlike physical mutagenesis, chemical mutagens insert point mutations through deamination, alkylation, and base modification, thereby creating wide range of genetic diversity. Among the commonly used chemical mutagens, EMS, NTG/MNNG, sodium azide, and nitrous acid have been widely used for strain improvement. EMS is the most frequently used alkylating agent for inducing GC → AT transitions, which results in a large amount of stable point mutations suitable for forward genetic screening. In comparison with physical mutagenesis, chemical mutagenesis generates large amount of point mutations, making them particularly useful for improving enzyme production using subtle mutations in metabolic and regulatory pathways. In *A. oryzae*, chemical mutagenesis has been successfully used to enhance the production of industrially important enzymes, including phytase and bioactive secondary metabolites. For instance, *A. oryzae* UJIIB-29 treated with EMS, sodium azide and nitrous acid produced mutants exhibiting up to 1.9-fold higher phytase activity than the parental strain (Haq et al. 2022). Similarly, mutagenesis of *A. oryzae* RP-21 with NTG produced mutants with a 2.4-fold increase in gluconic acid production, accompanied by improved glucose dehydrogenase and glucose oxidase activities (Raksha et al. 2012). Despite its effectiveness, chemical mutagenesis remains a random method requiring extensive phenotypic screening and subsequent molecular characterization to screen for beneficial mutations.

### Adaptive laboratory evolution

Adaptive Laboratory Evolution (ALE) offers another powerful classical strategy. By cultivating strains under sustained selective pressure, such as high cellulose load, low pH, osmotic stress, or toxin exposure, beneficial mutations accumulate across generations (Lubbers et al. 2019). ALE of *A. niger* generated strains with several fold increased cellulase secretion, while ALE of *A. oryzae* produced variants with strongly elevated inulinase activity (Lübeck and Lübeck 2022; Meyer et al. 2020). Although ALE is slow and non‑targeted as compared to genetic engineering, it can improve traits controlled by complex regulatory architectures that are difficult to optimize rationally. Consequently, ALE can be combined with random mutagenesis to accelerate the generation and screening of beneficial mutations during strain improvement. Iterative selection and screening rounds remain important even after mutagenesis or engineering, because improved producers must still be identified. Traditional screening methods involve plating on indicator media like starch iodine plates for amylase, or halo assays for protease or small-scale fermenters with enzyme assays. Once a hit is found, it is backcrossed or further mutated to accumulate improvements.

### Protoplast fusion

Protoplast fusion is a classical recombination-based strain improvement employed in modern biotechnology to recombine genomes with desirable traits in producer organisms to obtain superior strains for biotechnological applications. Intraspecific, interspecific and intergeneric protoplast fusion techniques have all been reported for improved production of pigment, β-glucosidase and L-asparaginase, respectively (Asitok et al. 2023; Goda et al. 2024; Zhu et al. 2010). By enzymatically removing cell walls and inducing PEG‑mediated fusion, researchers recombined genomes of different strains or different species to create hybrid strains. Unlike other mutagenic techniques, protoplast fusion enables simultaneous recombination of beneficial traits from various parental strains without requiring targeted genetic modification. A classic demonstration showed that fusion of *T. reesei* with *A. niger* generated a strain producing approximately three times more citric acid than the parental *A. niger* strain (El-Bondkly 2006; Salazar-Cerezo et al. 2023). Fusion of different *A. niger* strains has generated improved cellulase or amylase secretors. Intrageneric protoplast fusion was used for cellulase production in *Aspergillus* species. Protoplast fusion was conducted between *A. flavus* and *A. terreus*, *A. nidulans* and *A. tamarii,* and *A. oryzae* and *A. tubingensis*, with the resulting strains showing higher activity levels than the other strains (Goda et al. 2024). However, despite its effectiveness in generating superior hybrids, protoplast fusion still faces several bottlenecks, including low fusion efficiency, genetic instability of fusants, difficulty in selecting desirable recombinants, and limited reproducibility in large-scale industrial applications.

### Genome shuffling

Genome shuffling is a recombination-based approach used to improve industrially important microbial phenotypes. Genome shuffling is the recombination between multiple parents of each generation, and several rounds of genome fusion are carried out. Unlike conventional protoplast fusion, genome shuffling employs multiple rounds of fusion and screening among superior mutant strains, allowing beneficial mutations accumulation from various strains within a single genotype. The advantage of this technique is that the genetic breeding can be performed on the tested microbes without knowing their genetic background, making it a highly effective method (Gong et al. 2009; Li et al. 2014). This method accelerates the accumulation of beneficial mutations and has been applied to enhance cellulase, amylase, protease, and organic acid production. For example, genome shuffling applied to *Aspergillus glaucus* (*A. glaucus*) obtained strains with improved cellulase production (Zhao et al. 2014b). Similarly, combined mutagenesis, recursive protoplast fusion, and high throughput screening were used to improve transglycosylation activity in *A. niger* (Li et al. 2014). However, only a limited number of studies have investigated genome shuffling technology for improvement of transglycosylation activity. Its wider application in *Aspergillus* remains limited by various challenges, including efficient protoplast preparation and regeneration, maintenance of genetic and phenotypic stability, and validation of improved strains under industrially relevant fermentation conditions.

In summary, mutation-based strain improvement methods have produced many high yield *Aspergillus* strains in use today. The key characteristics of industrial strains fast growth, high secretory flux, and stress tolerance often arose from decades of classical breeding. Taken together, contemporary strain engineering increasingly relies on iterative integration of random diversification, high throughput phenotypic sorting, genomic deconvolution, and targeted reconstruction. These methods laid the foundation upon which modern molecular tools now build. Instead of being replaced, classical breeding methods are increasingly used as complementary approaches to modern genetic engineering by generating valuable mutations that can be subsequently characterized and optimized.

## Modern genetic engineering and synthetic biology

Recent advances in genetic engineering and synthetic biology have transformed *Aspergillus* species from traditionally improved industrial fungi into highly programmable microbial cell factories. Current state-of-the-art engineering strategies integrate CRISPR-based genome editing, synthetic promoter systems, pathway reconstruction, regulatory network rewiring, secretion engineering, chassis development, and systems metabolic engineering to enhance enzyme secretion, organic acid biosynthesis, heterologous protein expression, and strain robustness under industrial fermentation conditions (Jin et al. 2021; Sun et al. 2024).

Among these technologies, CRISPR/Cas systems have become the core platform for precise genome engineering in *Aspergillus* species. High efficiency CRISPR/Cas9 and CRISPR/Cas12a systems now enable targeted gene deletions, multiplex editing, promoter replacement, transcriptional regulation, and marker free genome modification in species including *A. oryzae*, *A. niger*, *A. terreus*, *A. nidulans*, and *A. sojae* (Gou et al. 2025; Modaffari et al. 2024; Sun et al. 2024). A series of chassis strains of *A. niger* (AnN1, AnN2) constructed and optimized for heterologous protein expression through CRISPR mediated deletion of the major glucoamylase genes and protease genes. They introduced multiple gene cassettes into the engineered chassis resulting in efficient heterologous protein production. AnN2 achieved higher titers for these diverse proteins in standard fermentation, far exceeding typical wild-type levels. This engineering of an *A. niger* demonstrates the effectiveness of CRISPR for heterologous protein production (Gou et al. 2025). Similarly, CRISPR mediated engineering of *A. oryzae* has enabled targeted optimization of secretion pathways, elimination of undesirable secondary metabolite biosynthetic clusters, and improvement of industrial enzyme productivity (Gou et al. 2025; Jin et al. 2022).

Synthetic biology has further enabled reconstruction and optimization of complex biosynthetic pathways in *Aspergillus* species. Multi-gene pathway assembly, modular cloning systems, and genome-scale metabolic rewiring are now routinely employed for the production of enzymes, organic acids, pharmaceuticals, and secondary metabolites (Sun et al. 2024). Engineering efforts in *A. terreus* have improved lovastatin and itaconic acid biosynthesis through central carbon metabolism optimization and pathway balancing (Hasan et al. 2021; Liu et al. 2024a). Advanced chassis engineering strategies have also generated mycotoxin-free industrial *A. oryzae* lineages through deletion of aflatoxin, cyclopiazonic acid, and other secondary metabolite gene clusters, significantly improving biosafety for food and biopharmaceutical applications. *Aspergillus* species are not only enzyme factories but also powerful producers of secondary metabolites (Lehmbeck et al. 2025). Metabolic rewiring has dramatically increased the production of kojic acid, penicillin, and organic acids. Notably, *A. oryzae* strains have been turned into kojic acid and penicillin producers by inserting or overexpressing biosynthetic genes. For example, Marui et al. increased penicillin yields 100-fold in *A. oryzae* by strong overexpression of *pcbAB* and *pcbC* with the *gpdA* promoter. Similarly, strains of *A. oryzae* for kojic acid have been constructed by expressing its oxidoreductase and transporter genes (Marui et al. 2010; Zhang et al. 2024). These examples highlight that even native secondary metabolites can be ramped up by modern engineering.

Beyond precise genome editing, further improvements in industrial phenotypes increasingly rely on engineering transcriptional and regulatory network engineering. Synthetic promoter libraries, tunable expression systems, and transcription factor manipulation are now widely used to optimize metabolic flux and secretion capacity. Regulatory proteins such as *AmyR*, *XlnR*, and *CreA* have been extensively engineered to improve expression of amylases, xylanases, cellulases, and other CAZymes. Promoter engineering using strong constitutive or inducible promoters, including *gpdA*, *amyB*, and *glaA*, has substantially enhanced heterologous protein expression and pathway balancing in industrial strains (Gila et al. 2022; Umemura et al. 2020; Zheng et al. 2025).

Another important frontier in engineering *Aspergillus* species involves secretion pathway and organelle engineering. Manipulation of ER-associated folding systems, vesicle trafficking, chaperone co-expression, protease gene deletion, and stress-response pathways has significantly improved extracellular protein secretion and strain stability (Sakekar et al. 2021; Schalén et al. 2016; Yoon et al. 2011). Engineering of proteostasis networks and glycosylation pathways is also becoming increasingly important for therapeutic protein production and industrial enzyme maturation (Rubio et al. 2016). Recent work at Novonesis (formerly Novozymes) has yielded an *A. oryzae* lineage cleared of mycotoxin genes (Lehmbeck et al. 2025). By naturally isolating a variant with a large chromosomal deletion removing aflatoxin and cyclopiazonic acid gene clusters, and then deleting the 3-nitropropionic acid genes, researchers created a strain that cannot make any of the three known *A. oryzae* mycotoxins. They also inactivated the penicillin gene cluster to remove any unintended antibiotic production. Strains of this lineage now produce a variety of recombinant proteins including GRAS approved bovine β-lactoglobulin producing strain, without safety concerns. This is a powerful example of precision breeding, rather than random mutagenesis, in which genome analysis guided the removal of entire toxin pathways (Lehmbeck et al. 2025).

Systems biology and multi-omics technologies now play a central role in state-of-the-art *Aspergillus* engineering. Comparative genomics, transcriptomics, proteomics, metabolomics, and fluxomics are increasingly integrated with machine learning and genome scale metabolic models to identify engineering targets and predict metabolic bottlenecks. These methods allow rational improvement of carbon utilization, energy utilization, and secretion capability, and tolerance to stress in industrial fermentation environments. At the same time, ALE in combination with genome resequencing and CRISPR based reconstruction has become a powerful tool for identifying beneficial mutations associated with higher production and adaptation to environment (Gou et al. 2025; Meyer et al. 2020; Wang et al. 2023).

Despite these major advances, several challenges still limit the advancement for industrial engineering of *Aspergillus* species. The filamentous growth morphology complicates genetic manipulation, clonal isolation, and large scale screening (Meyer et al. 2016). Unlike other unicellular microorganisms, *Aspergillus* species undergo complex development from spores to highly branched multinucleate hyphal networks, followed by sporulation for reproduction. This filamentous hyphal network results in heterogeneous fungal cell populations, variable growth morphology, and difficulties in maintaining genetic homogeneity during strain engineering and industrial fermentation. Morphology dependent rheology, oxygen transfer limitations, heterokaryon formation, and strain instability remain major barriers during industrial scale fermentation (Itani et al. 2025; Kitamoto 2015; Wollborn 2024). Additional challenges include off target genome editing, variable efficiency among *Aspergillus* species, limited availability of regulatory genetic elements, and difficulties with multiplex genome engineering (Jin et al. 2022). Furthermore, balancing high secretion capacity with cellular fitness and metabolic burden remains a key challenge in fungal cell factory design.

Overall, the integration of CRISPR based genetic engineering, synthetic biology, metabolic engineering and AI assisted design is rapidly advancing *Aspergillus* species towards the highly programmable fungal chassis for sustainable bio-industrial production. These advances in high-throughput genome engineering are expected to further accelerate development of robust strains capable of efficient production of enzymes, organic acids, pharmaceuticals, and high value biochemicals (Li et al. 2025; Yang et al. 2025; Zhang et al. 2023a).

## High-throughput screening strategies

Efficient strain improvement requires screening large numbers of variants. Current high throughput screening (HTS) platforms for *Aspergillus* species can be broadly classified into droplet-based microfluidics, biosensor-based screening, fluorescence activated cell sorting (FACS), automated robotic screening and image based phenotypic analysis, each with its advantages for rapid screening of superior strains (Jansen et al. 2021; Müller et al. 2022). In recent years, HTS technologies have been adapted to *Aspergillus* species. A breakthrough is the use of droplet-based microfluidics in which individual fungal spores or small hyphal fragments are encapsulated in nanoliter droplets with growth media and fluorescence reporter and sorted based on optical density or secreted enzyme activity. Consequently, HTS has become crucial part of modern strain engineering by enabling rapid evaluation of large mutant libraries generated using classical mutagenesis, CRISPR based genome editing, and adaptive laboratory evolution.

Beneyton et al. pioneered droplet based microfluidic screening for *A. niger*, demonstrating that picolitre scale droplets are unsuitable because hyphal tips rapidly exit the compartment, causing droplet coalescence while enlarged nanoliter droplets successfully supported hyphal growth, retaining secreted α-amylase inside the droplets (Beneyton et al. 2016). This platform was subsequently employed for *A. oryzae,* enabling the screening of approximately 450,000 droplets and identification of mutants exhibiting 6.6-fold higher α-amylase secretion, followed by genome resequencing and targeted validation of beneficial mutations linked to morphology, protein secretion, and carbohydrate metabolism (Li et al. 2023b). This demonstrates how microfluidic HTS can drastically accelerate finding rare high‐producers. The combination of microfluidic compartmentalization, fluorogenic enzymatic assays, and dielectrophoretic fluorescence activated droplet sorting (DEP-FADS) represent a shift from plate-limited screening towards ultra-high throughput evolutionary engineering. DEP-FADS enables rapid sorting of droplets according to fluorescence intensity thereby allowing direct screening of hyperproducing mutants at single spore resolution. The platform maintained high sorting with an accuracy at four droplets per second with 100% positive droplets sorting and could operate at up to 21 droplets per second without destabilization (Baret et al. 2009; Beneyton et al. 2016; Fischlechner et al. 2014; Guo et al. 2021; Jian 2023; Zinchenko et al. 2014).

Furthermore, droplet based microfluidic screening approach dramatically reduces both screening time and cost as compared to conventional microtiter plate-based screening methods. A study quantified that 10^8^ individual enzyme reaction could be processed by ultrahigh throughput droplet microfluidics within 10 h using less than 150 µL of the total reaction volume, enabling a 1000-fold increase in screening speed and approximately one million fold in cost reduction as compared to conventional robot microtiter plate screening (Agresti et al. 2010). These capabilities fundamentally transformed *Aspergillus* strain improvement by enabling efficient screening of large mutant libraries generated through classical mutagenesis, CRISPR based engineering, and adaptive laboratory evolution. Rather than screening hundreds of samples, microfluidics allows hundreds of thousands of samples such as CRISPR libraries, UV/chemical mutant libraries, or adaptive evolution populations within realistic timeframes. Taken together, droplet-based microfluidic screening represents one of the most powerful HTS technologies now available for fungal biotechnology, delivering speed, precision, miniaturization, and the ability to couple phenotypic sorting with downstream deep sequencing and targeted editing (Li et al. 2025; Guo et al. 2024; Yang et al. 2025; Zhang et al. 2023a). Complementary screening platforms remain essential for validating enzyme activity, metabolic phenotypes, and production stability under industrial conditions.

In parallel, fluorescent or colorimetric biosensors are used to link enzyme activity to a detectable signal. For instance, substrates conjugated to fluorophores or chromophores are included in agar or liquid media: colonies that secrete high enzyme levels create bright halos. Some groups engineer transcriptional biosensors, such as reporter gene fusions to promoters induced by a target metabolite. While these are more common in bacteria, analogous systems are emerging in fungi. For *Aspergillus*, pH-sensitive dyes or fluorescent glycans can serve as readouts. Conventional chromogenic and fluorogenic substrates remain the most widely used biosensors for enzyme screening, while genetically encoded biosensors are still under development for fungi. Emerging biosensor technologies, such as extracellular split GFP are concepts currently being explored to further enhance throughput for fungal strains screening (Beneyton et al. 2016; Furukawa et al. 2007; Miller et al. 1998; Zhang et al. 2023a). Automated microtiter plate culturing using 96 or 384 well plates coupled with robotic handling and plate readers are widely used for medium throughput screening of *Aspergillus* strains. Colony picking robots can array thousands of colonies. Recent advancements in automated microscopy and AI-assisted imaging further enable quantitative assessment of fungal colony morphology, pellet formation, and growth kinetics provides additional phenotypic information for strain selection.

Among cell sorting technologies, FACS provides another valuable platform for fungal strain screening. Flow cytometry itself has limited use for fungi due to large hyphae, but can sort spores in germination. Recent methods combine FACS of spores with inducible expression, selecting for clones that later show high production in culture. As synthetic libraries of mutant gene knockouts, promoter variants, transcription factor over expressors become larger, HTS becomes essential to rapidly identify the best candidates (Li et al. 2023b). However, filamentous growth presents unique challenges, such as extension of hyphae beyond borders of droplets. Recent advancements in encapsulation strategies, viscosity adjustments, spore confinement and osmotic balance stabilization have made possible to achieve stable formation of microcolony in picolitre droplets.

## Conclusion and future perspectives

The journey of *Aspergillus* strain engineering reflects the evolution of industrial biotechnology itself. Classical strain improvement methods created high producing strains, while modern strain engineering enabled precision genome engineering and high throughput screening. Crucially, these approaches are synergistic. Rather than replacing classical approaches, modern genetic engineering, systems biology and synthetic biology complement classical strain engineering methods enabling precise identification, validation and optimization of useful mutations. The most effective results come from iterative random mutagenesis for mutant generation following genome sequencing for identification of mutations and CRISPR based modifications, and high throughput screening for identification of high performing strains. As technology advances, more sophisticated approaches will become feasible. Future strain improvement will rely on large scale combinatorial genetics by high throughput methods, effectively combining rational design with directed evolution.

Looking forward, synthetic and systems biology will drive the next wave of innovations. Regulatory and metabolic models will help guide rational edits. Integrating genome scale modeling, multi-omics, and machine learning could predict how to balance the cellular growth with protein secretion. Development of industrial chassis strains with synthetic minimal genomes having only essential genes represents a promising long-term direction, allowing dedicated platforms which are optimized for the production of certain proteins. Modern CRISPR tools, including prime editing and base editing, might allow genome engineering without introducing double strands breaks to minimize the cellular stress. Synthetic genetic circuits may also dynamically adjust the metabolic pathways to optimize the yield.

**Fig. 5:**
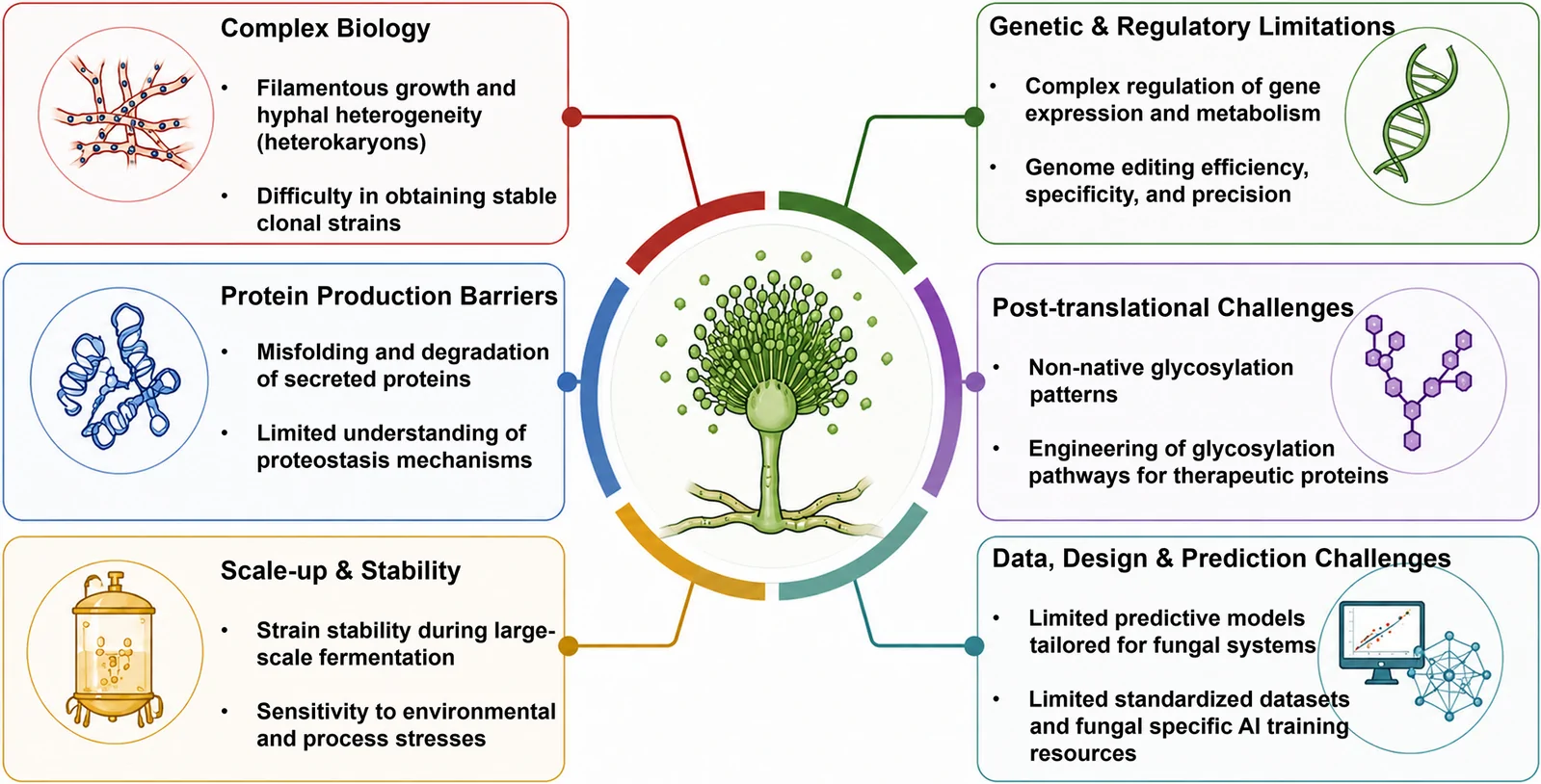
Summary of the future challenges in *Aspergillus* strain engineering. The figure summarizes interconnected challenges affecting the development of industrial *Aspergillus* strains: Complex Biology, including filamentous growth, hyphal heterogeneity, and difficulties in obtaining stable clonal strains; Protein Production Barriers, including misfolding and degradation of secreted proteins and limited understanding of proteostasis; Scale-up and Stability, including maintenance of strain performance during large scale fermentations and sensitivity to environmental and process stresses; Genetic and Regulatory Limitations, including complex regulation of gene expression and metabolism and constraints on genome editing; Post Translational Challenges, including non-native glycosylation patterns and the need for glycosylation pathway engineering for applications requiring defined protein glycoforms; and Data, Design, and Prediction challenges, including limited fungal specific predictive models, standardization datasets, and resources for AI-assisted strain engineering. AI: Artificial intelligence

Despite these advancements, future challenges still remain (Fig. 5). The filamentous nature of *Aspergillus*, its hyphal growth pattern, and the presence of heterokaryons complicate the process of achieving engineered clonal strains. Secreted proteins can degrade or misfold, making the manipulation of chaperone systems and the proteostasis pathways an ongoing research frontier. For therapeutic protein production, glycosylation patterns often need humanization, necessitating deep pathway engineering. Additionally, ensuring stability of the strain in large scale fermentation remains a critical concern. Addressing these challenges necessitates integrated advances in fungal genetics, systems biology, process engineering and computational design.

In summary, *Aspergillus* strain engineering is transitioning into a more advanced era where classical methods converge with cutting edge biological tools. The next generation of strains will likely result from integrating iterative evolutionary approaches with designed synthetic constructs. By integrating genome engineering approaches with next generation high throughput screening platforms will enable rapid navigation of vast genetic design spaces and accelerate strain engineering. We anticipate that *Aspergillus* will remain at the forefront of industrial biotechnology as continued integration of genome engineering, synthetic biology, systems biology, artificial intelligence and high throughput screening enables the development of fully programmable fungal cell factories that rival or exceed nature’s most efficient hyper producers.

## Data Availability

All information discussed in this review are available from the cited published literature.

## Declarations

### Declaration on the use of generative AI

The authors used a generative AI tool (ChatGPT, OpenAI) to develop the initial concepts for the illustrative figures and to assist with language polishing of the manuscript. All AI-assisted content was subsequently edited, reviewed, and verified by the authors. The authors take full responsibility for the final content of the manuscript.
